# Systemic Immune-Inflammation Index and Mortality in Testicular Cancer: A Systematic Review and Meta-Analysis

**DOI:** 10.3390/diagnostics13050843

**Published:** 2023-02-22

**Authors:** Farley E. Salazar-Valdivia, Valeria A. Valdez-Cornejo, Juan R. Ulloque-Badaracco, Enrique A. Hernandez-Bustamante, Esteban A. Alarcón-Braga, Melany D. Mosquera-Rojas, Diana P. Garrido-Matta, Percy Herrera-Añazco, Vicente A. Benites-Zapata, Adrian V. Hernandez

**Affiliations:** 1 Escuela de Medicina, Universidad Peruana de Ciencias Aplicadas, Lima 15023, Peru; 2 Sociedad Científica de Estudiantes de Medicina de la Universidad Peruana de Ciencias Aplicadas, Lima 15023, Peru; 3 Sociedad Científica de Estudiantes de Medicina de la Universidad Nacional de Trujillo, Trujillo 13011, Peru; 4 Grupo Peruano de Investigación Epidemiológica, Unidad para la Generación y Síntesis de Evidencias en Salud, Universidad San Ignacio de Loyola, Lima 15012, Peru; 5 Escuela de Medicina, Universidad Privada San Juan Bautista, Lima 15067, Peru; 6 Universidad Privada del Norte, Trujillo 13011, Peru; 7 Unidad de Investigación para la Generación y Síntesis de Evidencias en Salud, Vicerrectorado de Investigación, Universidad San Ignacio de Loyola, Lima 14072, Peru; 8 Unidad de Revisiones Sistemáticas y Meta-análisis, Guías de Práctica Clínica y Evaluaciones de Tecnología Sanitaria, Vicerrectorado de Investigación, Universidad San Ignacio de Loyola, Lima 15012, Peru; 9 Health Outcomes, Policy, and Evidence Synthesis Group, University of Connecticut School of Pharmacy, Mansfield, CT 06269, USA

**Keywords:** testicular cancer, meta-analysis, overall survival, progression free survival, systemic immune-inflammation index

## Abstract

The systemic immune-inflammation index (SIII) is a marker studied in multiple types of urologic cancer. This systematic review evaluates the association between SIII values with overall survival (OS) and progression-free survival (PFS) in testicular cancer. We searched observational studies in five databases. The quantitative synthesis was performed using a random-effects model. The risk of bias was assessed using the Newcastle–Ottawa Scale (NOS). The only measure of the effect was the hazard ratio (HR). A sensitivity analysis was performed according to the risk of bias in the studies. There were 833 participants in a total of 6 cohorts. We found that high SIII values were associated with worse OS (HR = 3.28; 95% CI 1.3–8.9; *p* < 0.001; I^2^ = 78) and PFS (HR = 3.9; 95% CI 2.53–6.02; *p* < 0.001; I^2^ = 0). No indication of small study effects was found in the association between SIII values and OS (*p* = 0.5301). High SIII values were associated with worse OS and PFS. However, further primary studies are suggested to enhance the effect of this marker in different outcomes of testicular cancer patients.

## 1. Introduction

According to GLOBOCAN, 74,458 new testicular cancer cases and 9334 deaths were estimated in 2020, of which 33.7% were in Europe and 27.7% were in Asia. However, Asia has a wide advantage regarding mortality, with a rate of 42.8% [1]. The worldwide incidence of these tumors has more than doubled in the last 40 years [2]. By 2025, an increase in incidence is anticipated for Europe, Latin America, some parts of Asia, and even in areas where the incidence is relatively low [3,4]. This type of cancer is the most common in young adult men between the ages of 15 and 34 years, representing 1.5% of male neoplasms and 5% of urological tumors, in general.

Testicular cancer can be classified, according to histopathology, into germ cell tumors and non-germ cell tumors, of which germ cell tumors account for 98% [5]. The former, in turn, are classified as seminomas, which are the most common in adults and patients with cryptorchidism or non-seminomas [6]. Genetic and environmental factors will influence the increase in incidence. Various diseases, including Down syndrome and testicular dysgenesis syndrome, are linked to an increased risk of testicular cancer [7]. Thanks to the substantial advances in the treatment of testicular cancer in recent decades, this is the most curable solid malignancy [8,9].

The literature reports various risk factors related to a poor prognosis in testicular cancer. Among the most studied factors, age over 35 years, serum alpha-fetoprotein above 1000 Ku/L before chemotherapy, and human chorionic gonadotropin above 5000 IU/L stand out [10]. Likewise, an interval between orchiectomy and the start of chemotherapy of fewer than three weeks, high-volume metastatic load, and the treatment site are reported [10].

Recently, the association between a poor cancer prognosis and different inflammatory markers has been described. A study using systemic inflammatory markers, based on preoperative complete blood count, found that neutrophils, the neutrophils to lymphocytes ratio (NLR), and the mean red blood cell distribution width were significantly higher in the tumor group. In contrast, the mean volumes of platelets and lymphocytes were significantly higher in the cancer-free group [11].

As well as these markers, the NLR, the platelets to lymphocytes ratio (PLR), the monocytes to lymphocytes ratio (MLR), and the preoperative albumin to globulin ratio have been identified [12,13]. In 2014, the systemic immune-inflammation index (SIII), defined as SIII = P × N/L, was used for the first time, using lymphocyte (L), neutrophil (N), and platelet (P) counts, thus providing a strong predictive factor for the prognosis of patients with hepatocellular carcinoma [14]. Its use in various cancers is currently being investigated; however, regarding its use in testicular cancer, it is believed that SIII can obtain more robust data than routine markers on staging and cancer prognosis, knowing that a high SIII reflects a worse prognosis [15]. Due to the evidence for the use of SIII in testicular cancer, these results should be combined to provide clinicians with a more reliable tool. This study aimed to evaluate the association between SIII and survival outcomes in testicular cancer.

## 2. Methods

### 2.1. Research Question and Study Design

We used the PECO strategy: population (P), exposure (E), comparison (C), and outcome (O) to guide the main objective of this systematic review. Based on the PECO strategy, we ask the following question: Do patients with testicular cancer (P) and high values of SIII (E) have worse overall survival and progression-free survival (O) than patients with testicular cancer and low values of SIII (C)?

### 2.2. Register and Report Guideline

We registered the study protocol on the International Prospective Register of Systematic Reviews (PROSPERO). The register code is CRD 42021281533. Likewise, we used the Preferred Reporting Items for Systematic Reviews and Meta-analysis (PRISMA) statement (see the PRISMA checklist in Supplementary Table S1) [16] and the Cochrane Handbook of Systematic Reviews [17].

### 2.3. Search Strategy and Data Sources

A comprehensive literature search was performed by searching the following databases: PubMed, Scopus, Web of Science, Embase, and Cochrane Library. In addition, a manual search was performed in pre-print platforms, such as MedRxiv and Scielo preprints, with the purpose of including as many articles as possible within our study related to the studied subject. The search strategy was developed using the Peer Review of Electronic Search Strategies (PRESS) checklist [18]. No language restrictions were applied.

### 2.4. Eligibility Criteria, Study Selection, and Data Extraction

We included studies that: (i) evaluated the association between the systemic immune-inflammation index (SIII) and testicular cancer reporting outcomes, such as progression-free survival (PFS) and overall survival (OS), (ii) included males with a confirmed diagnosis of testicular cancer, and (iii) items that provide a defined cut-off value for the systemic immune-inflammation index (SIII) (see the inclusion and exclusion criteria of the patients for each study in Supplementary Table S2).

To manage the data, we used the Rayyan QCRI software (Rayyan Systems Inc.©, Cambridge, MA, USA) to help in the screening and selection of studies [19]. Four authors (FES-V, VAV-C, JRU-B, and EAH-B) independently screened the titles and abstracts of the retrieved records. The titles and abstracts that met the inclusion/exclusion criteria went on to the next phase of the selection process. Then, we independently assessed the remaining records using the full text of each study. Any conflict in the selection process was resolved by reaching a consensus among all the authors. Finally, all the data from the selected studies were extracted by the authors (FES-V, VAV-C, JRU-B, and EAH-B) with a special data collection card in a standardized file in Microsoft Excel and imported into the Mendeley platform for reference purposes. The entire study selection is shown in the PRISMA flow chart [16].

We collected the following data: author, year, country, median follow-up time, participants’ median/mean age, the type of testicular cancer, cut-off values, and associations between systemic immune-inflammation index values and overall survival or progression-free survival (see definitions of the outcomes for each study in Supplementary Table S3).

### 2.5. Quality Assessment

The quality of the study was assessed using the Newcastle–Ottawa Scale (NOS) for case-control/cohort studies, by two authors (FES-V and VAV-C), based on 3 different items: selection, comparability, and outcome/exposure [20]. The maximum that can be achieved in a study is 9 points; a score ≥7 points was considered a low risk of bias; otherwise, ≤6 points was considered a high risk of bias. Any disagreement was resolved by reaching a consensus among all authors.

### 2.6. Data Synthesis and Publication Bias

The information collected from the selected articles was combined using Review Manager 5.4 (RevMan 5.4) (The Cochrane Collaboration, Copenhagen, Denmark). Hazard ratios (HRs) and 95% confidence intervals (CIs) were the only measures used to obtain a pooled effect. We used a random-effects model (DerSimonian and Laird) for the quantitative analysis. The heterogeneity of the studies was evaluated using Cochran’s Q test with a *p*-value of <0.1 and an I^2^ statistic with values >70% as a sign of severe heterogeneity. A sensitivity analysis was performed, excluding articles with low methodological quality, to test the robustness of our findings. Egger’s test was carried out to assess publication bias; *p*-values less than 0.1 indicated publication bias [21,22].

## 3. Results

### 3.1. Research Question and Study Design

**Figure 1:**
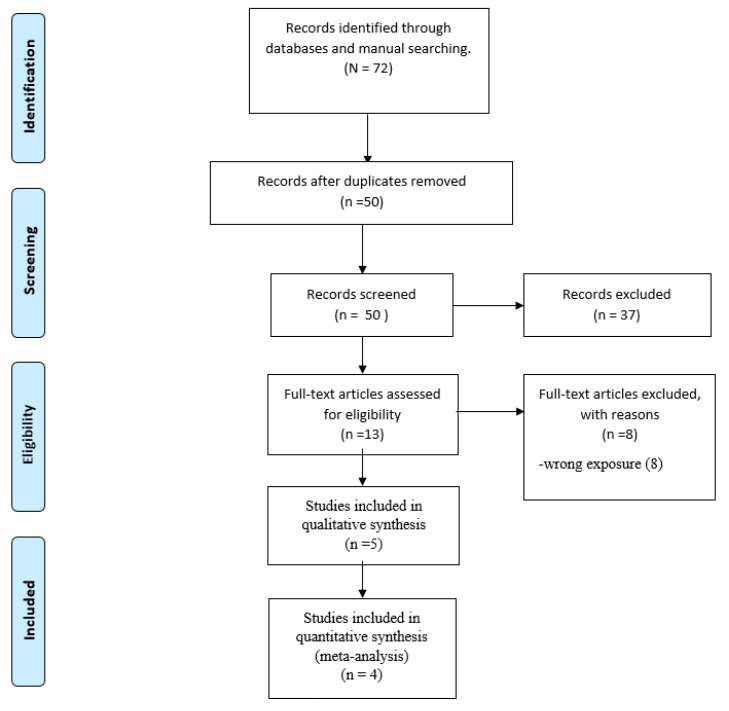
PRISMA flow diagram.

We identified 72 articles, leaving 50 studies after eliminating duplicates. The screening process evaluating titles and abstracts left 13 studies for full-text review. Likewise, the full-text screening left five studies that met all the selection criteria [23,24,25,26]. Figure 1 summarizes the study selection process.

### 3.2. Study Characteristics

Four studies were included from a total of five cohorts since the study by Chovanec et al. [25] analyzed two cohorts. All cohorts analyzed OS, and only three cohorts analyzed PFS. In addition, two cohorts were conducted in Slovakia, one in Turkey, one in China, and one in Japan. The included studies were conducted between 2017 and 2021. The total number of participants was 833. The age of the participants ranged from 16 to 84 years. The most frequent type of testicular cancer was germ cell tumor, and the median follow-up time ranged between 39.2 and 63.4 months. Four cohorts evaluated the optimal SIII cut-off values for OS and PFS, ranging from 719 to 1428. The details of the included articles are summarized in Table 1. In the quality assessment of the included articles, four cohort studies had a low risk of bias, whereas only one study had a high risk of bias due to problems in cohort comparability on the basis of design or analysis and follow-up that was not long enough for the outcomes to occur (Supplementary Table S4).

### 3.3. Association between SIII Values and OS in Testicular Cancer

The association was assessed in five cohorts (n = 805). However, only four studies were included in the meta-analysis (the study by Frankhauser CD et al. [26] was not included in the meta-analysis because they reported an association HR per 10-fold increase (log10 [HR]: 30.2, 95% CI 3–304; *p* < 0.05, which could alter and bias our results).

**Figure 2:**
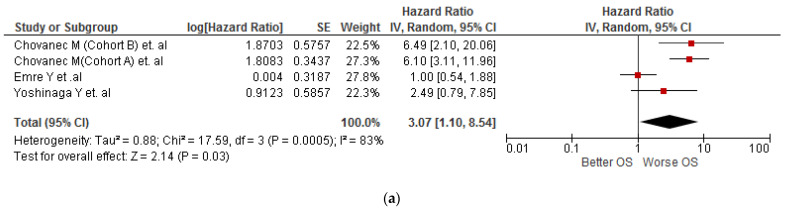

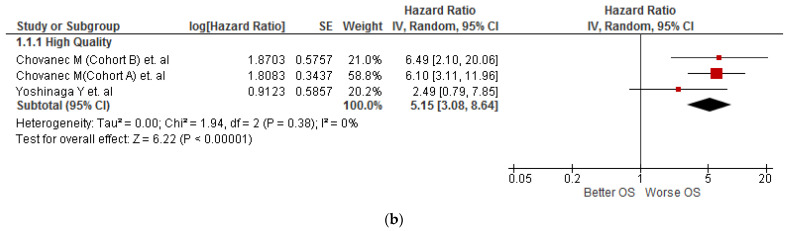
(**a**) Association of SIII values and OS in patients with testicular cancer [23,24,25]. (**b**) Sensitivity analysis according to the risk of bias of the association between SIII values and OS in patients with testicular cancer [24,25].

In the meta-analysis, we found that testicular cancer patients with high SIII values were associated with worse OS (HR: 3.07; 95% CI 1.1–8.54; *p* < 0.001; I^2^ = 83) (Figure 2a). Due to the severe heterogeneity, a sensitivity analysis was performed where only studies with a low risk of bias were included, where the association was maintained. (HR: 5.15; 95% CI 3.08–8.6; *p* < 0.001) but with null heterogeneity (I^2^ = 0%) (Figure 2b).

### 3.4. Association between SIII Values and PFS in Testicular Cancer

**Figure 3:**
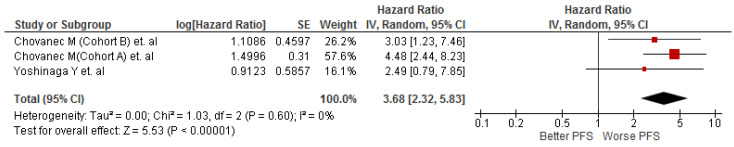
Association of SIII values and PFS in patients with testicular cancer [24,25].

The association was assessed in four cohorts (n = 443). We found that testicular cancer patients with high SIII values were associated with worse PFS (HR: 3.68; 95% CI 2.32–5.83; *p* < 0.001) with null heterogeneity (I^2^ = 0) (Figure 3).

### 3.5. Publication Bias

Publication bias could not be determined because there were no more than five meta-analyzed studies in any of the outcomes.

## 4. Discussion

Our main results show that a high SIII value is associated with worse OS and PFS in patients with testicular cancer. This reflects the role of inflammation in the genesis and progression of several types of cancer, including urological cancers.

In tumor genesis, inflammation mediates the creation of reactive oxygen species and the activation of cell signaling pathways that promote cell proliferation and limit apoptosis [27]. In the progression of malignancy, it influences the cellular components of the immune system, and its chronic state stimulates immunity and is associated with a poor prognosis [28,29]. In this sense, several studies have demonstrated the importance of some inflammatory markers in the prognosis of cancer patients, such as the lymphocyte/monocyte ratio, which is a prognostic indicator in head and neck cancer [30], rectal cancer [31], ovarian cancer [32] and cholangiocarcinoma [33]. We also have the NLR, which is a prognostic indicator in melanoma [34], endometrial cancer [35], or solid tumors [36]. Similarly, the PLR can be used in rectal cancer [37], gastric cancer [38], cholangiocarcinoma [39], and head and neck cancer [40].

As a tumor marker, SIII reflects the balance between host inflammation and immune response status [41]. Likewise, it reflects systemic inflammation in a more balanced manner and has a higher predictive value than other inflammatory markers in patients with cancers [42,43]. Several studies have shown the role of SIII as a prognostic marker in cancers. In this regard, the role of SIII was shown to predict worse OS in patients with colorectal cancer, breast cancer, hepatocellular carcinoma, small cell lung cancer, acral melanoma, and gastric and esophageal cancer [44,45,46,47,48]. Although the reasons are not entirely clear, it is suggested that it has to do with the individual effect of the SIII components. SIII is based on peripheral neutrophil, platelet, and lymphocyte count. Therefore, high SIII corresponds to high platelet/neutrophil and/or low lymphocyte counts [44].

In the case of neutrophils, their presence is associated with poor prognosis in cancer patients because they can activate endothelial and parenchymal cells that facilitate metastasis of circulating tumor cells [49]. In addition, neutrophils also mediate cancer cell proliferation and metastasis by secreting inflammatory mediators and angiogenic proteins that participate in tumor cell proliferation, migration and invasion, and tumor immunosuppression in the stages of carcinogenesis [50,51,52,53,54,55].

On the one hand, platelets can protect circulating tumor cells from the antitumor immune response and promote the angiogenesis and metastasis of cancer cells [56]. In vitro, platelets also release various growth factors that enhance cancer cell proliferation [57]. Likewise, platelets can also recruit and activate granulocytic cells in tumor tissues, and thus, may be essential for generating tumor-associated neutrophils [58,59,60,61].

Moreover, lymphocytes, especially tumor-infiltrating lymphocytes, play a key role in the host’s immune response to cancer [62]. They can also induce cell death and inhibit tumor cell proliferation and migration [63]. Lymphocytes are responsible for the adaptive immune response and participate in cancer immunosurveillance and immunoediting [64]. Lymphopenia usually indicates the severity of the disease; it helps cancer cells escape from the immune system of tumor-infiltrating lymphocytes, whose formation is related to the process of lymphocyte migration to the tumor microenvironment, so a decreased level of tumor-infiltrating lymphocytes predicts worse survival in cancer patients [65]. Although no studies have been conducted in this regard, there is no reason why, to date, these mechanisms suggested in cancers, in general, should not also be considered for application to patients with testicular cancer.

Several systematic reviews evaluating the prognostic role of SIII in urological cancers have been published. A systematic review of 15 articles found, in subgroup analyses, that high SIII indicated a worse overall survival rate in urinary cancers and hepatocellular carcinoma, gastrointestinal tract cancers, small cell lung cancer, and acral melanoma [44,45,46,47,48]. Another systematic review of 22 articles showed that SIII over the cutoff value could predict worse overall survival in urinary system cancer, hepatocellular carcinoma, gastric cancer, esophageal squamous cell carcinoma, small cell lung cancer, non-small cell lung cancer, and acral melanoma [66]. From the combined data of 13 papers, it was found that a high pre-treatment SIII indicated markedly worse OS, PFS, and cancer-specific survival [67]. Finally, from the pooled results of 14 studies, it was found that high SIII was associated with worse OS in patients with urologic cancers. Patients with high SIII values also had poorer PFS and cancer-specific survival as well as lower OS than patients with low SIII values. In addition, the subgroup analysis of OS and PFS showed that the prognosis of patients with high SIII was worse than that of patients with low SIII [68]. However, only one included a study of patients with testicular cancer [15]. This systematic review of 12 studies with 2693 patients included the study by Yang J et al. [23], which is also part of our study and contributed 28 patients to that review. The authors found that a high SIII value was associated with worse OS, PFS, and cancer-specific survival rates in patients with various urological cancers, including bladder, renal cell, and prostate carcinoma [12]. Therefore, our study would be the first systematic review evaluating the association between SIII patients’ survival outcomes in testicular cancer.

Our results show the potential role of SIII in survival outcomes of patients with testicular cancer and being an easy-to-measure and low-cost marker. However, this biomarker, in comparison with other inflammatory markers used to predict the prognosis of urological cancers, such as NLR [69], PLR [70], and C-reactive protein/albumin ratios [71], contains three types of peripheral blood inflammatory cells, simultaneously [72]. This characteristic is relevant, as it better reflects the balance between inflammation and the body’s immune response, so it could be a better marker [42,43]. This is a hypothesis that needs to be corroborated in further studies. Furthermore, even though it was not explicitly evaluated for testicular cancer, in other systematic reviews, the association between a high SIII value and OS and PFS in patients with urologic cancers did not vary significantly according to tumor subtypes, cancer stages, sample sizes, study types, treatment methods, NOS scores, or a cut-off point defining elevated SIII [12]. This last point is relevant considering the wide range of SIII values in the studies included in our systematic review, like other reviews on the prognostic value of this biomarker in urologic cancers [65,66,67]. However, it is necessary to consider the ethnic component in assessing this biomarker since some studies show that the European population is more sensitive to this marker than the Asian population [10]. Additionally, the sensitivity analysis considering only the studies with a low risk of bias, allowed us to reaffirm our results. Finally, the applicability of SIII in clinical practice remains conceptual due to flaws in the design of studies. Most studies are retrospective with different cut-off points, measurement time points, and chosen endpoints, and although many studies adjusted their analysis for various factors, unmeasured confounders cannot be excluded [73]. Regarding meta-analyses that evaluated its usefulness, the retrospective design and the heterogeneity between the studies that make them up, limit the strength of this type of study, and the sensitivity analysis often alters the results obtained in a first evaluation [73]. Consequently, even though it is a promising marker, we cannot state that it is better than others that are available to assess the prognosis of these patients, which merits further studies on this topic.

In our study, we found limitations that should be considered. Firstly, due to the small number of studies, it was impossible to perform a correct stratification of results according to the clinical or sociodemographic variables of the patients. Secondly, it was only possible to study the association of SIII values with two clinical outcomes, so it would be necessary for future studies to study the association with other outcomes. Third, high statistical heterogeneity was found due to the methodological and clinical differences between the studies. However, heterogeneity decreased when the sensitivity analysis was performed, which only included a low risk of biased studies. Finally, sensitivity and specificity values of the SIII cut-off points in OS and PFS have not been reported, which could help evaluate the precise prognostic value of this biomarker in testicular cancer.

## 5. Conclusions

High SIII values are associated with worse OS and PFS. However, further primary studies are suggested to enhance the effect of this marker in different outcomes of testicular cancer patients.

## Figures and Tables

**Table 1:** Characteristics of the included studies.

Author	Year	Country	Median Follow-Up Time	Participants	Median/Mean Age (IQR/SD)	Type of Testicular Cancer	Outcome	HR (95% CI), *p*-Value	Cut-Off
Emre Y et al. [23]	2021	Turkey	55 months	244	38 (10)	Mixed	Overall Survival	1.004 (0.5376–1.875), *p* = 0.99	719
Frankhauser CD et al. [26]	2018	Switzerland	53 months	146	34 (9)	Mixed	Overall Survival	30.2 (3–304), *p* < 0.05 ^a^	1428
Yoshinaga Y et al. [24]	2021	Japan	63.4 months	63	35 (16–67)	Germ cell tumor	Overall Survival	4.87 (0.59–40.47), *p* = 0.14	NR
							Progression free-survival	2.49 (0.79–7.85), *p* = 0.12	
Chovanec M (Cohort A) et al. [25]	2017	Slovakia	49 months	171	30 (17–62)	Germ cell tumor	Overall Survival	6.1 (3.11–11.95), *p* < 0.05	1003
							Progression free-survival	4.48 (2.44–8.23), *p* < 0.05	
Chovanec M (Cohort B) et al. [25]	2017	Slovakia	85 months	181	30 (16–67)	Germ cell tumor	Overall Survival	6.49 (2.1–20.03), *p* < 0.05	1003
							Progression free-survival	3.03 (1.23–7.46), *p* < 0.05	

NR, not reported; ^a^, After log10 transformation: the HR thus corresponds to a 10-fold increase in the variable.

## Data Availability

Not applicable.

## Supplementary Materials

The following supporting information can be downloaded at: https://www.mdpi.com/article/10.3390/diagnostics13050843/s1, Table S1: PRISMA checklist, Table S2. Inclusion and exclusion criteria for patients in the studies, Table S3: Outcome definitions of included studies; Table S4: Newcastle–Ottawa quality assessment scale for included studies. Table S5: Search Strategy.
